# Current insights into the regulation of programmed cell death by TP53 mutation in cancer

**DOI:** 10.3389/fonc.2022.1023427

**Published:** 2022-10-13

**Authors:** Yali Su, Yingying Sai, Linfeng Zhou, Zeliang Liu, Panyan Du, Jinghua Wu, Jinghua Zhang

**Affiliations:** ^1^ Department of Clinical Laboratory, North China University of Science and Technology Affiliated Tangshan Maternal and Child Heath Care Hospital, Tangshan, China; ^2^ Department of Clinical Laboratory, North China University of Science and Technology Affiliated Hospital, Tangshan, China

**Keywords:** TP53 mutation, ferroptosis, pyroptosis, apoptosis, autophagic cell death, cancer

## Abstract

Gene mutation is a complicated process that influences the onset and progression of cancer, and the most prevalent mutation involves the TP53 gene. One of the ways in which the body maintains homeostasis is programmed cell death, which includes apoptosis, autophagic cell death, pyroptosis, ferroptosis, NETosis, and the more recently identified process of cuprotosis. Evasion of these cell deaths is a hallmark of cancer cells, and our elucidation of the way these cells die helps us better understands the mechanisms by which cancer arises and provides us with more ways to treat it.Studies have shown that programmed cell death requires wild-type p53 protein and that mutations of TP53 can affect these modes of programmed cell death. For example, mutant p53 promotes iron-dependent cell death in ferroptosis and inhibits apoptotic and autophagic cell death. It is clear that TP53 mutations act on more than one pathway to death, and these pathways to death do not operate in isolation. They interact with each other and together determine cell death. This review focuses on the mechanisms *via* which TP53 mutation affects programmed cell death. Clinical investigations of TP53 mutation and the potential for targeted pharmacological agents that can be used to treat cancer are discussed.

## TP53 and programmed cell death in cancer

TP53 is a tumor suppressor gene that plays an important role in the cell cycle, DNA repair, and cell senescence and apoptosis (1). This gene codes for the p53 protein, which is a transcription factor that prevents abnormal proliferation and division of cells (2). Recent research has found that TP53 is involved in the regulation of various types of programmed cell death (PCD), which include apoptosis (3), autophagy (4, 5), pyroptosis (6), and ferroptosis (7), and pathways for generation of reactive oxygen species (ROS) (8–10). For example, p53 promotes apoptosis by upregulating apoptosis-related proteins in order to stabilize the organism (11, 12). However, mutant p53 has the reverse effect on PCD.

**Figure 1:**
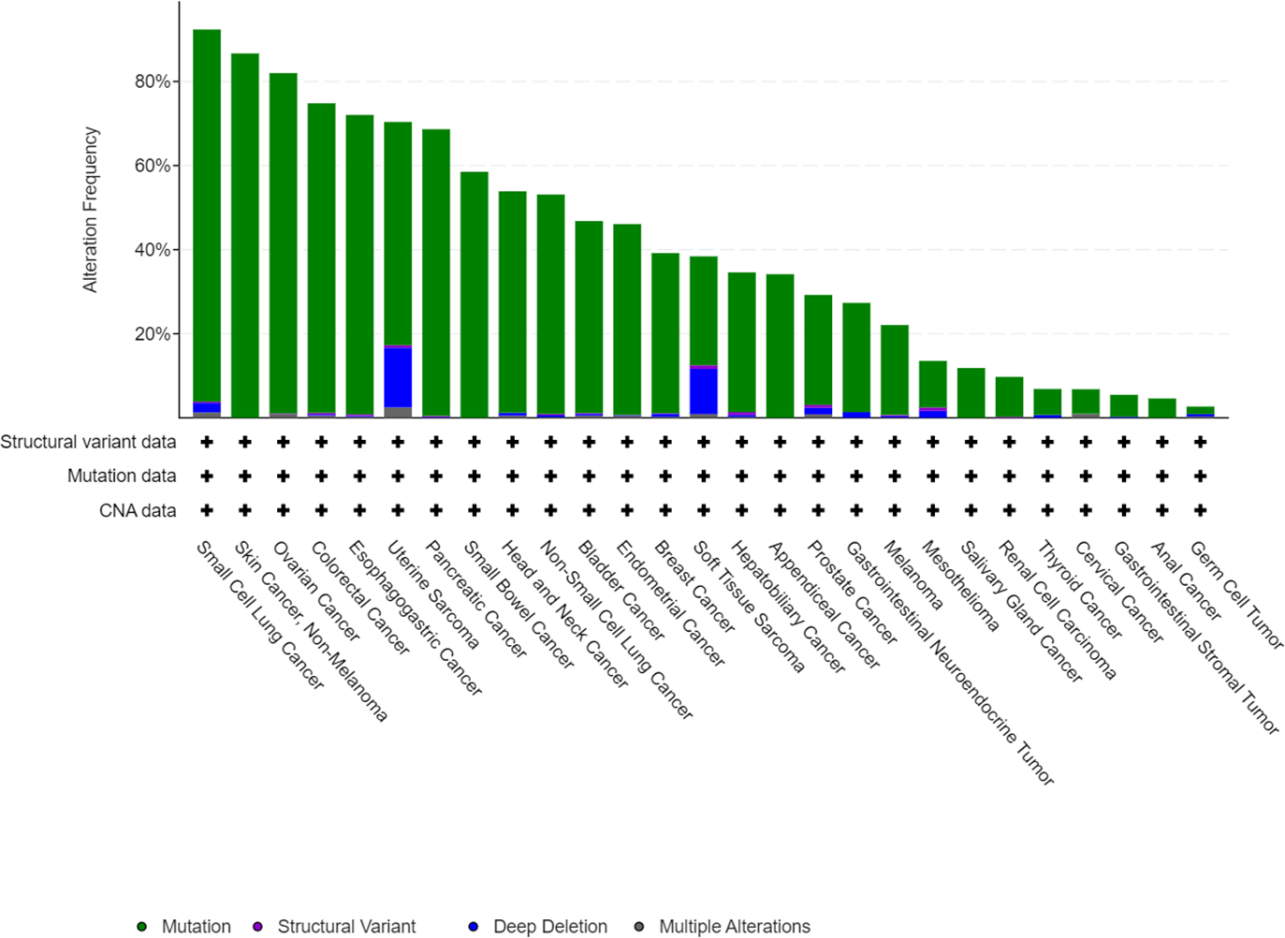
Mutation features of TP53 in tumors identified in the MSK MetTropism database on the cBioPortal website. The symbol "+" indicates the number of samples corresponding to the cancer analysis, which is identified in detail with the cBioPortal website.

Normal p53 protein is degraded rapidly. However, when transcribed by an mutated TP53 gene, p53 accumulates in tumor cells, accelerating the development and progression of cancer (13). TP53 mutation has been identified in approximately half of all cancers, and analysis of the MSK MetTropism database on the cBioPortal website shows that the mutation frequencies vary according to the type of cancer (**Figure 1**) (14, 15). It has been confirmed that the most common location for mutation of TP53 is in amino acid residues 102–292 of its DNA binding domain (16). These mutations are usually highly expressed in malignant cells and produce three carcinogenic properties: loss of p53 function, resulting in inability to activate downstream target genes; dominant negative effects (DNE), resulting in blocking the function of normal p53 protein in cells; and acquisition of new functions that usually promote tumor development (17). In brief, mutated TP53 loses its normal function as a tumor suppressor gene and becomes an oncogene instead (13). In recent years, there have been many discoveries of new ways of death, and many studies have clarified the mechanism of these modes of death in cancer. At the same time, there are more and more in-depth studies on TP53 gene mutations in tumors, but there is no discussion on how TP53 mutations regulate programmed cell death. Therefore, this paper will explore the relationship between TP53 mutation and programmed death, and provide new ideas about the treatment of tumors.

## TP53 mutation in cancer

**Figure 2:**
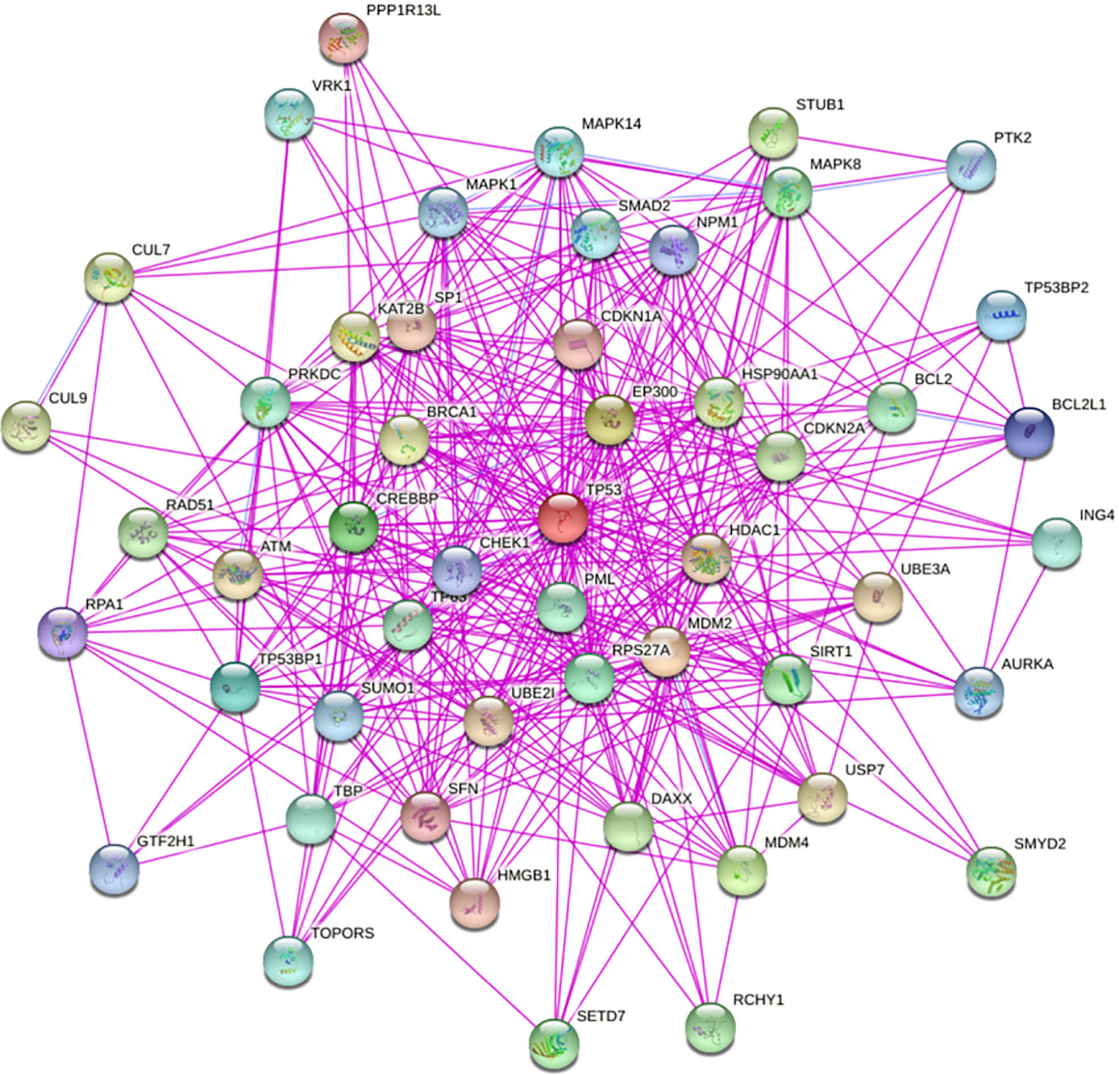
We obtained TP53-binding proteins through the STRING website (https://string-db.org/).

**Figure 3:**
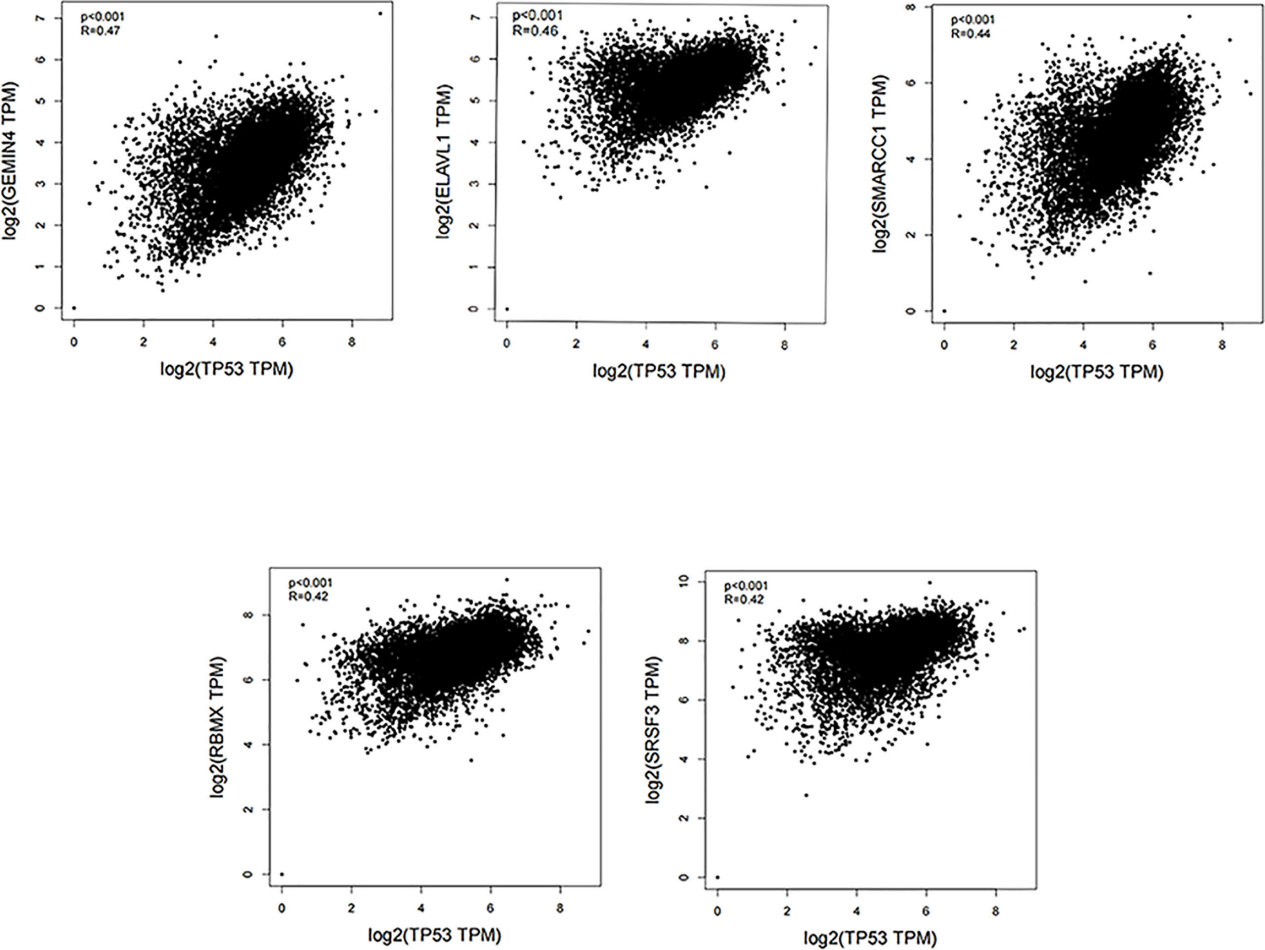
Using the GEPIA2 approach, we compiled the top 100 TP53-related targeting genes in The Cancer Genome Atlas database of cancers. We also investigated the relationship between TP53 and each of the top five genes (GEMIN4, ELAVL1, SMARCC1, RBMX, and SRSF3) using Pearson’s correlation method.

The TP53 gene is located on the short arm of chromosome 17 and consists of 11 exons, the role of which is to encode the p53 protein (18). The p53 protein is normally present at a low level. However, p53 levels have increased when DNA is damaged by environmental stimuli or spontaneous replication errors, oncogenes and ROS are activated. Normal (wild-type) p53 performs a range of biological tasks, including regulating the cell cycle, repairing damaged DNA, controlling target gene transcription, stabilizing the genome, and regulating numerous biological processes, including angiogenesis, apoptosis, metabolism and cell senescence (19–21). These diverse biological functions reflect the large number of genes regulated by p53. It is estimated that p53 directly regulates about 500 target genes (22). Using the TP53-binding proteins listed on the STRING website (https://string-db.org/) (**Figure 2**) and our basic settings are as follows: minimum required interaction score [“Low confidence (0.150)”], meaning of network edges (“evidence”), max number of interactors to show (“no more than 50 interactors” in 1st shell) and active interaction sources (“experiments”). The instructions for interactive gene expression profiling analysis outlined on the GEPIA2 website (http://gepia2.cancer-pku.cn/#analysis), we have now compiled a list of the top 100 TP53-related targeting genes from The Cancer Genome Atlas database. We then investigated the relationship between TP53 and each of the top five genes (GEMIN4, ELAVL1, SMARCC1, RBMX, and SRSF3) using the Pearson’s correlation coefficient (**Figure 3**).

The most common mutations in the TP53 gene are missense mutations in its DNA binding domain. Although these missense mutations affect only one amino acid, they can have a significant impact on the p53 protein (23). For example, some p53 variants result in a new functional phenotype that can promote tumor development by inactivating tumor proteins p63 and p73 (24, 25). Furthermore, mutations in the TP53 gene can be used as a biomarker of cancer and to monitor the prognosis and guide treatment. For example, recent studies have shown that somatic mutations in TP53 (p53-R273H) play a key role in chemotherapy-induced colorectal cancer stem cells. It is also proved that p53-R273H mutation enhances colorectal cancer stemness through regulating specific lncRNAs (26). Moreover, studies have also found that somatic mutation of TP53 (p53-R248Q) functions as an oncogene in promoting endometrial cancer by up-regulating REGγ. Therefore, REGγ is a promising therapeutic target for endometrial cancer with the p53-R248Q mutation (27).TP53 mutations tend to be associated with a poorer prognosis in cancer because the mutant p53 they produce often leads to loss of apoptotic function and the ability to arrest the cell cycle.

## Programmed cell death and cancer

PCD has an important role in maintaining dynamic homeostasis in multicellular organisms. During both normal development and aberrant physiological conditions and diseases, PCD removes cells that are at risk of becoming cancerous and those that have been invaded by pathogens (28). Several distinct types of PCD have been identified, including apoptosis, necroptosis (a regulated type of necrosis), autophagy, pyroptosis, ferroptosis, NETosis, and, most recently, cuproptosis (29–33). In these modes of death, apoptosis, pyroptosis and necroptosis seem to be related to each other through the caspase family. A study has shown that caspase-1 can initiate apoptosis in the absence of gasdermin D (34). Caspase-1 is an enzyme that produces the classical pathway of pyroptosis. The activated caspase-1 plays a role by cleaving Gasdermin D (**Figure 6**). This study shows that cells can initiate apoptosis through caspase-1 in the absence of pyroptosis. In addition, a study has shown that caspase-8 can inhibit necroptosis mediated by RIPK3 and MLKL (35). This study demonstrates that the occurrence of apoptosis will inhibit the occurrence of necroptosis.Autophagic cell death appears to be associated with NETosis, because it has been shown that mTOR regulates NET formation by posttranscriptional control of expression of hypoxia-inducible factor 1 α (HIF-1α) (36).

At present, cancer is a common cause of premature death. A study predicts that the number of cancer patients worldwide will increase to the next 50 years, twice the number estimated to have been diagnosed in 2018 (37). Therefore, human research on cancer has never stopped. Therefore, clarifying the mechanism of programmed cell death can provide more possibilities for clinical treatment of cancer. For example, some studies have found that Schizandrin A can inhibit the proliferation of non-small cell lung cancer and breast cancer cells by inducing apoptosis (38, 39). Meanwhile, Badgley et al. (40) found that deletion of SLC7A11 induced tumor-selective ferroptosis and inhibited Pancreatic ductal adenocarcinoma growth.

## TP53 mutation and PCD

### Role of TP53 mutation in ferroptosis

**Figure 4:**
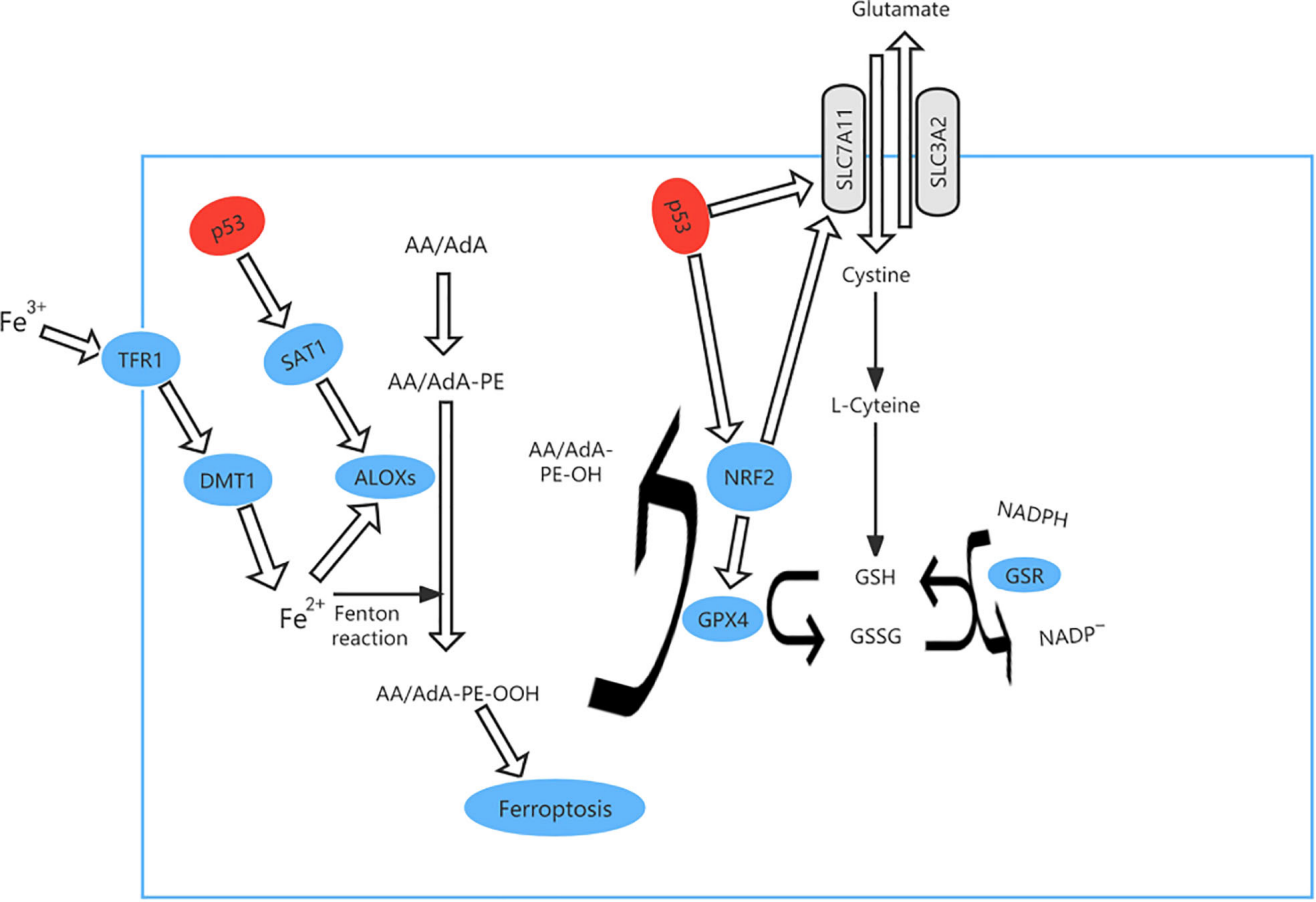
The ferroptosis pathway and role of p53 in ferroptosis. p53 can promote iron-dependent cell death via transcriptional inhibition of expression of solute carrier family 7 member 11 (SLC7A11) expression. At the same time, TP53 controls transcription of GPX4 and SLC7A11 via nuclear factor erythroid 2-related factor 2 (NRF2). p53 can also mediate the expression of arachidonate 15-lipoxygenase (ALOX15) by inducing spermidine/spermine N1-acetyltransferase 1 (SAT1), thereby promoting iron-dependent cell death.

Ferroptosis is an iron-dependent form of PCD that is triggered by deactivation of. the lipid repair enzyme glutathione peroxidase 4 (GPX4) and loss of glutathione reductase, resulting in a low level of reduced glutathione and accumulation of lipid peroxides, which react with ferrous ions to generate ROS (**Figure 4**). Iron is a potentially toxic nutrient that is regulated by p53 to maintain homeostasis. Several studies have demonstrated that p53 can promote iron-dependent PCD *via* transcriptional inhibition of the expression of solute carrier family 7 member 11 (SLC7A11, which is a transmembrane protein and a component of the cystine-glutamate antiporter (xCT) (41–44). p53 can also mediate expression of arachidonate 15-lipoxygenase by inducing spermidine/spermine N1-acetyltransferase 1, thereby promoting iron-dependent PCD (45).

**Figure 5:**
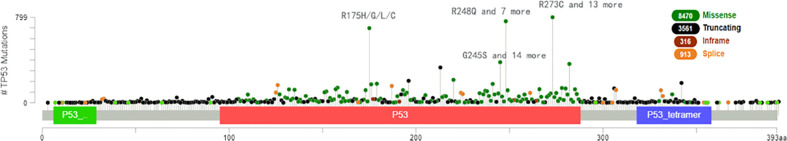
The most clinically prevalent TP53 mutation by the MSK MetTropism database on the cBioPortal website.

TP53 mutation causes loss of the ability to destroy tumor cells and is found in approximately 50 percent of human cancers (46) and ferroptosis can be accelerated when acetylation of TP53 is lost (47, 48). For example, p53^3KR^ can suppress tumor growth and regulate the metabolic target in cells by direct combination of TP53 with the promoter, which stops transcription of xCT and promotes ferroptosis (49). We analyzed the most clinically prevalent TP53 mutation by the MSK MetTropism database on the cBioPortal website, as shown in **Figure 5**. In small cell lung cancer cell lines, the representative cell lines with five mutations (R248Q, R273H, R175H, G245S, or R249S) were found to be more ferroptosis-sensitive than a wild-type TP53 cell line (46, 50). The genetic mutation may cause p53 to lose tumor-inhibiting activity of the wild-type protein (LOF, loss of function) or gain new properties (also known as functional gain, GOF) (51). Nonsense mutation, frameshift mutation or homozygous deletion may cause the function of p53 to be lost (52). Tumour cells have lost inhibitory activity due to a lack of expression of the p53 protein. Missense mutations frequently lead to new functions acquired for p53. In contrast, gain-of-function (GOF) causes tumor cells to overaccumulate mutated p53 protein.These mutated p53 proteins not only eliminate the tumor inhibitory function of wild-type p53, but also endow mutant proteins with new activities, which can actively promote all stages of tumor progression and increase resistance to anticancer therapy (53). In esophageal cancer and small cell lung cancer, TP53 controls transcription of GPX4 and SLC7A11 *via* nuclear factor erythroid 2-related factor 2 (NRF2), which is sensitive to drug-induced oxidative stress (54) and regulates ferroptosis by controlling the transcriptional expression of GPX4 and SLC7A11 (55).

The available evidence indicates that targeting ferroptosis may be an effective strategy for destroying cancer cells with TP53 mutation. There has been a report indicating that a combination of sulfasalazine and radiotherapy has a positive effect on tumor cells with mutation or other abnormality of TP53 (56). In another study, a combination of xCT blockade and administration of eprenetapopt (APR-246) destroyed esophageal cancer cells with mutant p53 (p53-R273H and p53-R175H) (49).

### Role of TP53 mutation in pyroptosis

**Figure 6:**
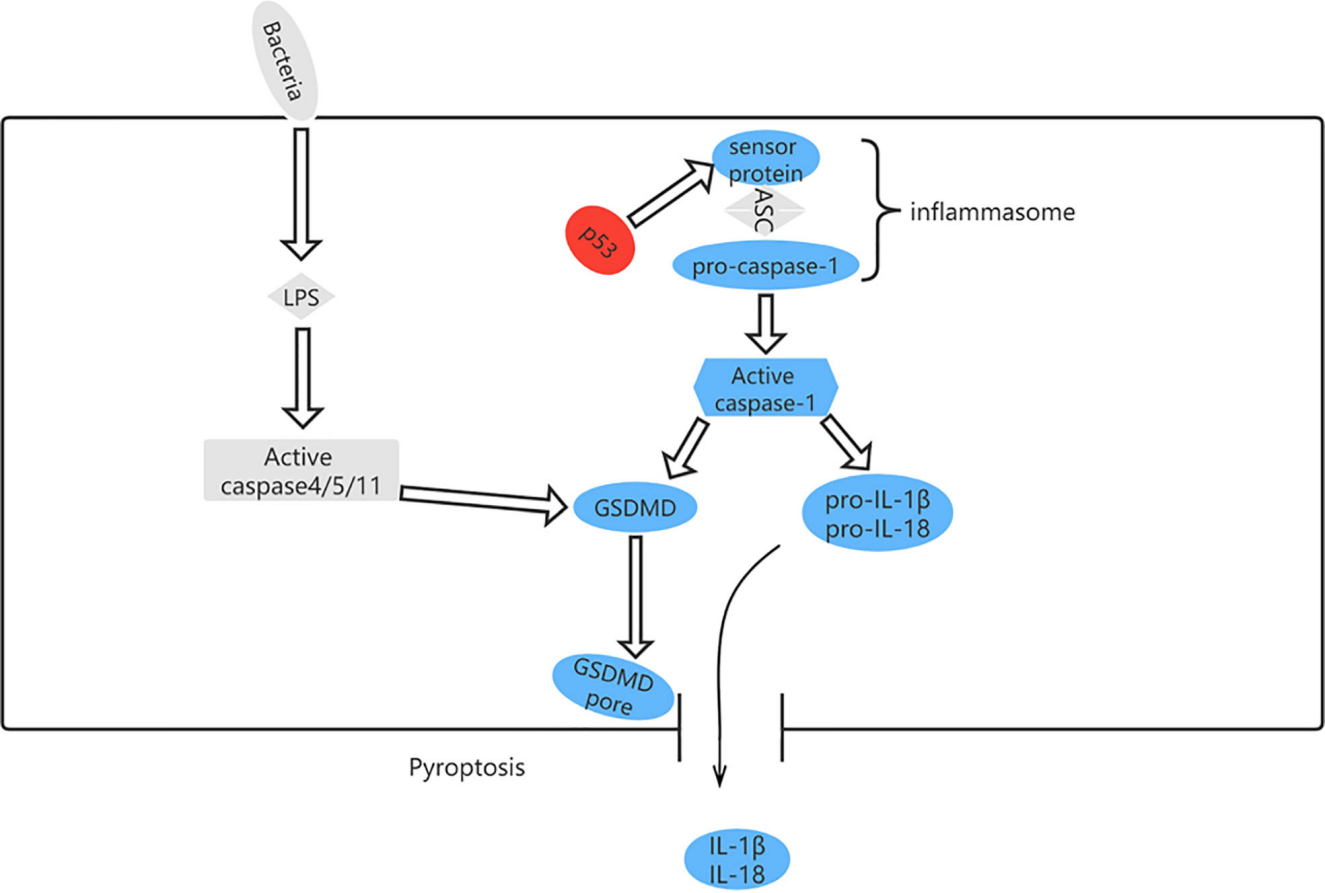
The pyroptosis pathway and role of p53 in pyroptosis. p53 can control pyroptosis by directly inducing transcription of the Ipaf (NLRC4) gene.

Pyroptosis can occur *via* a classical pathway or a non-classical pathway (**Figure 6**). In the classical pyroptosis pathway, pro-caspase-1 forms an inflammasome by combining apoptosis-associated speck-like protein with the inflammatory corpuscle receptors NLRP1, NLRP3, NLRC4, and AIM2 in response to infection. The inflammasome then activates precursors of caspase-1 to produce active caspase-1. Caspase-1 has two purposes: first, to cleave Gasdermin D (GSDMD) into a 31-kDa GSDMD-N fragment, which further mediates pyroptosis, and a 22-kDa GSDMD-C fragment; and second, to recruit, activate, and release proinflammatory cytokines, such as interleukin (IL)-1β and IL-18, to mount an extracellular inflammatory response (57, 58).

Pyroptosis occurs in many diseases and regulates cell death *via* a mechanism involving inflammation (59). However, it has both advantages and disadvantages in terms of tumorigenesis. On the one hand, pyroptosis is an innate immune mechanism that is able to suppress development and progression of cancer; on the other, it can promote inflammation and provide a microenvironment conducive to tumor growth (60). Although there is scant evidence to suggest that the mechanism of tumor inhibition is related to induction of pyroptosis in previously normal cells undergoing transformation to tumor cells, release of IL-1β and IL-18 can lead to infiltration and activation of immune cells at tumor sites, thereby supporting the anti-tumor immune response (61). Nevertheless, pyroptosis signaling pathways release inflammatory mediators that promote proliferation of tumor cells, angiogenesis, metastasis, DNA damage, and epigenetic changes (62–64).

Although pyroptosis differs from other types of cell death in mechanism (65), there is some evidence indicating a positive correlation between TP53 and pyroptosis in non-small cell lung cancer (66). Moreover, other studies have shown that high expression of GSDMD and GSDMC is associated with decreased progression-free survival in patients with serous ovarian cancer carrying TP53 mutation (67). p53 induces transcription of the Ipaf (NLRC4) gene, which activates caspase-1, indicating that TP53 is associated with pyroptosis (68). Comparison of the expression levels of 51 pyroptosis-related genes between normal and malignant tissues in The Cancer Genome Atlas data set revealed that expression of TP53 was upregulated. In pyroptosis-related diseases, the most frequent mutation is in TP53, followed by NLRP3 (69).

In light of the above findings, we can infer that TP53 mutation and pyroptosis have a complex relationship, the study of which will expand our understanding of tumor behavior and generate novel treatment strategies.

### Role of TP53 mutation in apoptosis

**Figure 7:**
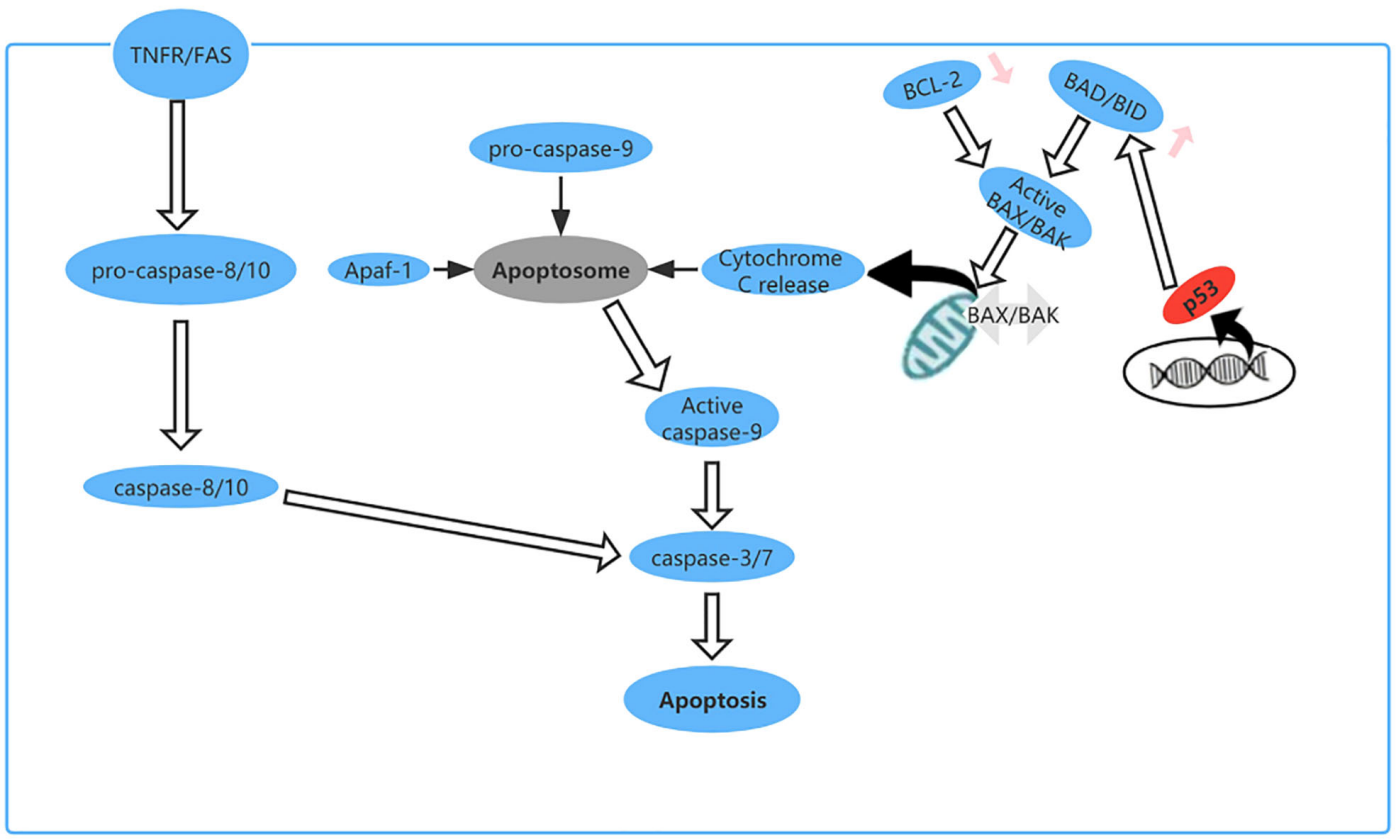
The apoptosis pathway and the role of p53 in apoptosis. p53 upregulates expression of BH3-only proteins (Bid, Bim, Bad, Bmf, Bik/Nbk, Blk, Noxa, Bbc3, and DP5) at the transcriptional level.

Apoptosis is a type of PCD that maintains homeostasis when cells are damaged by disease or harmful substances (70). For example, in cancer, apoptosis can destroy tumor cells and prevent growth and metastasis of tumors (71). As a tumor suppressor gene, TP53 plays an important role in regulating apoptosis. Cells subjected to cytotoxic or radiation-induced stress sense this external pressure *via* kinases and then phosphorylate or acetylate p53 protein at various locations, resulting in activated p53 (72), which upregulates expression of BH3-only proteins (including Bid, Bim, Bad, Bmf, Bik/Nbk, Blk, Noxa, Bbc3, and DP5) at the transcriptional level (73). These upregulated BH3-only proteins directly activate BAX/BAK, leading to formation of oligomers on the mitochondrial membrane, which increase the permeability of the outer mitochondrial membrane and cause release of cytochrome c into the cytoplasm. Cytochrome c binds to apoptosis protease activator factor (Apaf)-1, which recruits procaspase-9 for formation of apoptotic bodies. In the apoptosome, activation of caspase-9 by autoproteolytic cleavage initiates the caspase cascade pathway (29, 74) (**Figure 7**).

When TP53^R175H^, mutation occurs, the mutant p53 produced can interact with and inhibit caspase-8, caspase-9, and caspase-3 to compromise caspase-dependent apoptosis (75–77). There is some research showing that in non-small-cell lung cancer (NSCLC) TP53^R175H^ can induce expression of miRNA-128-2, which acts on E2F5 after transcription, resulting in loss of its inhibitory effect on transcription of p21 (waf1) in the cytoplasm and prevention of an anti-apoptotic effect by cleavage of caspase-3 (78). Another investigation revealed that in ovarian cancer some TP53 mutations, including TP53^P72R^, do not dramatically reduce the apoptotic activity of p53 (79). Thomas et al. also found that the P72R p53 mutant is structurally wild-type and exhibits the same affinity for the DNA sequence recognized by p53 (80). Furthermore, single nucleotide polymorphism in the TP53 gene may cause variation in both Arg72 and Pro72 (81). However, the Arg72 variant has been shown to be more effective in inducing apoptosis (82). It has also been found that p53 mutants cause resistance to the apoptosis induced by cisplatin, doxorubicin, and 5-fluorouracil by coordinating the miRNA network (78). In addition, some drugs work by restoring wild-type activity in cells with mutated TP53 or by inhibiting MDM2, which is a key negative regulator of TP53 (73).

### Role of TP53 mutation in autophagic cell death

**Figure 8:**
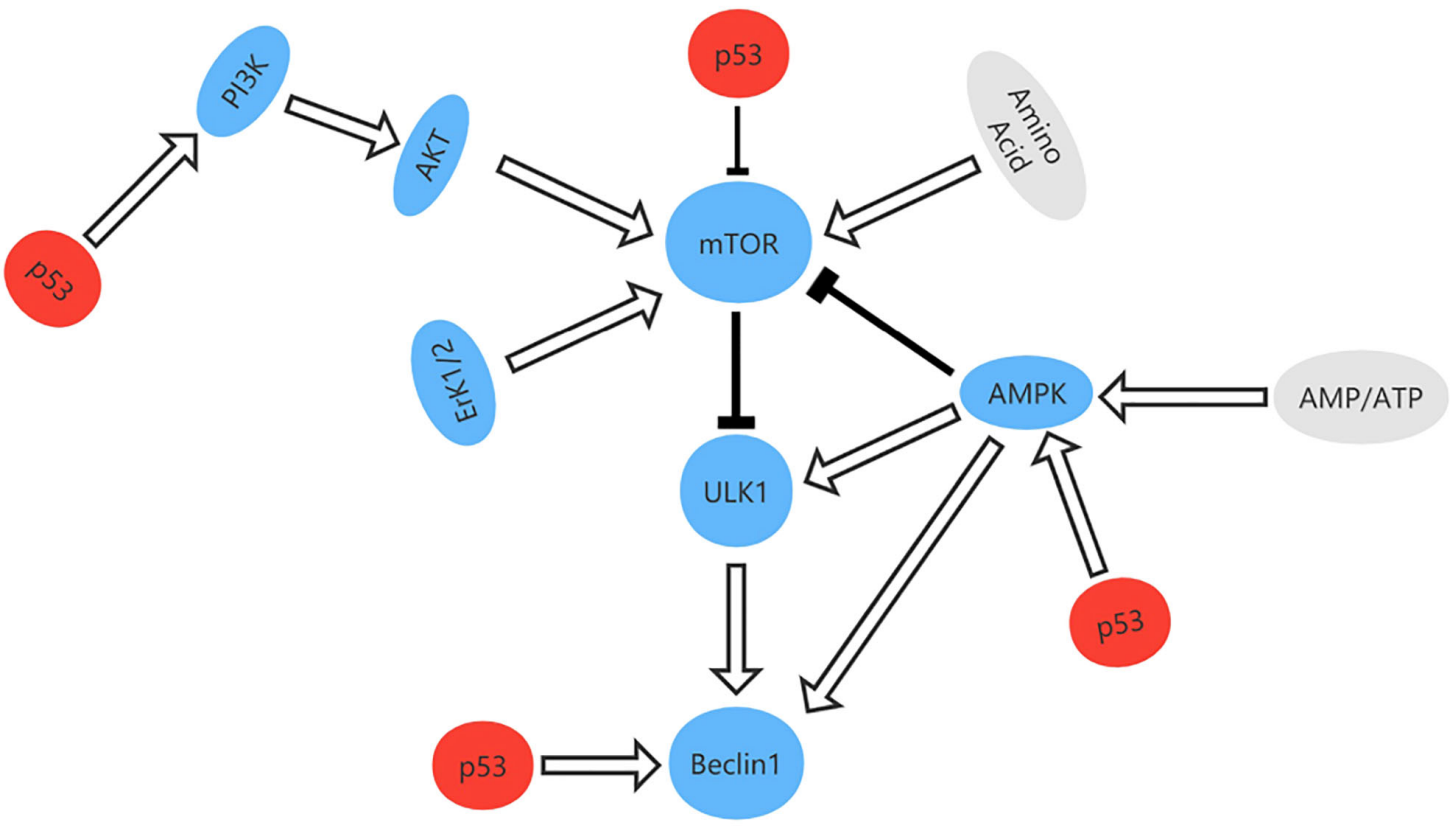
The signal pathway that regulates autophagy and the role of p53 in autophagy. p53 can activate the AMPK-TSC1/TSC2 and PI3K/Akt pathways to inhibit mammalian target of rapamycin (mTOR) and promote autophagy.

Autophagy is a degradation process that removes injured organelles and abnormally folded proteins *via* lysosomes and is associated with tumorigenesis and progression of cancer (83, 84). When deactivated, autophagy can protect tissues from chronic injury and prevent cancer. However, autophagy can also provide the energy needed by tumor cells, which helps to render them insensitive to hypoxia, depletion of nutrients, and immune stimulation therapy therapy (85). TP53 is thought to regulate autophagy and it has been shown that p53 can activate the AMPK-TSC1/TSC2 and PI3K/Akt pathways to inhibit mammalian target of rapamycin (mTOR) and promote autophagy (86, 87). Other targets of TP53 with an impact on autophagy include damage-regulated autophagy modulator (DRAM), which is a type of lysosomal protein generated by TP53. However, mutation of TP53 is one of the hallmarks of cancer. In pancreas and breast cancer cells, one study found that mutant p53 inhibits key autophagy-related proteins and enzymes, such as Beclin-1, ATG12, and AMPK, while also inhibiting the formation of autophagic vesicles by stimulating mTOR. Mutant p53 can also inhibit autophagy by activating continuous PI3K Akt/mTOR signaling (88, 89) (**Figure 8**).

According to the definition of autophagy, accumulation of mutant p53 with abnormal folding and partial denaturation can activate autophagy, which indicates that agents that activate autophagy could destroy tumor cells (90). Garufi et al. identified a novel type of zinc complex that degrades p53^R175H^ protein by autophagy (91). Furthermore, Shin et al. confirmed that DHA stimulates autophagy by increasing the lipidated form of LC3B in prostate cancer cells with mutant p53 (92).

### Role of TP53 mutation in NETosis

In 1996, Takei et al. found that neutrophil suicide under chemical stimulation by phorbol 12-myristate 13-acetate was different from that observed in necrosis or apoptosis (93). In this process, neutrophils can form extracellular structures known as neutrophil extracellular traps (NETs), which are composed of chromatin, nuclear histones, neutrophil proteins with antibacterial properties, and mitochondrial DNA and help to trap and destroy invasive microorganisms in the extracellular environment (94, 95). During this process, neutrophils first recognize PAMP and DAMP and then activate protein kinase c and nicotinamide adenine dinucleotide phosphate oxidase (NADPH oxidase, NOX), leading to generation of ROS (96). ROS then trigger activation of myeloperoxidase-mediated neutrophil elastase (97), which promotes decondensation of chromatin (97, 98). Chromatin swelling then causes rupture of the nuclear envelope and plasma membrane, releasing NET to capture and destroy pathogenic factors (99). Research has also shown that NET induces caspase-1-dependent pyroptosis in macrophages (100). Moreover, Amulic et al. (101) established that cyclin-dependent kinases 4 and 6, which are core members of the cell cycle mechanism, are necessary for formation of NETs. TP53 is known to regulate pyroptosis, inflammation, and the cell cycle, but there is no corresponding research on whether TP53 is related to NETosis.

### Role of TP53 mutation in immunogenic cell death

Recent years have seen emergence of the concept of immunogenic cell death, which has opened up new possibilities for the treatment of cancer. It is known that the body’s immune response can destroy tumor cells. The principle is that tumor antigens are presented to T-cells by dendritic cells, and the efficacy of anticancer therapy is reflected in the types and numbers of immune cells (102). However, the tumor cells that are destroyed also have immunogenicity, and dead autologous tumor cells can be used to create vaccines for adjuvant treatment of cancer (103). Some studies have found that TP53 is also involved in immune regulation. P53 can induce expression of transporter-associated antigen processing-1 and endoplasmic reticulum amino peptidase-1, which deliver tumor-associated antigens to the antigen-presenting cell membrane, resulting in the antigen-associated major histocompatibility complex being recognized by T-cells (104). However, mutated p53 can be recognized as a new antigen by T-cell receptors, rendering TP53 mutants sensitive to immunotherapy (105).

### Role of TP53 mutation in cuprotosis

Copper is an essential co-factor but can become toxic when its concentration exceeds the threshold, which suggests that cancer cells can be destroyed by increasing the concentration of copper in target cells (106). Recent studies have found that accumulation of lipoylated protein and loss of the Fe-S cluster protein result in copper-induced cell death in response to toxic stress (107). However, it is unclear whether copper-induced cell death is associated with TP53 mutation.

#### Role of TP53 mutation in necroptosis and paraptosis

Necroptosis is also a form of programmed cell death, which is independent of caspase and morphologically similar to necrosis. Necroptosis is activated by tumor necrosis factor receptor (TNFR) and Toll-like receptor (TLR)-4 or TLR3.Upon activation, these receptors recruit the adapter proteins Fas-associated death domain (FADD), TNF receptor-associated DD (TRADD), and TIR domain-containing adapter-inducing interferon-β(TRIF), which interact with receptor-interacting protein kinase 1 (RIPK1) and caspase-8 or-10. Ultimately they activate the necroptosis executioner mixed lineage kinase domain-like (MLKL) (29).Some studies have found that when mutant TP53 (p53-R273H orp53R175H) cells are surrounded by normal epithelial cells, mutant TP53 cells undergo necroptosis and are basally extruded from the epithelial monolayer. This shows that the necroptosis of TP53 mutant cells is caused by competition with surrounding normal cells (108).Furthermore, it has been found that progesterone (P4) can induce necroptosis in p53-deficient fallopian tube (FT) cells through the TNF-a/RIPK1/RIPK3/MLKL pathway (109).These studies show that necroptosis occurs in TP53 mutated cells, but the specific mechanism needs further study.

Paraptosis is a programmed cell death that is morphologically different from apoptosis and autophagy. It exhibits cytoplasmic vacuolation, independent of caspase, mitochondrial and endoplasmic reticulum swelling, but no pyknosis (110). Although the molecular mechanism of paraptosis is not clear, studies have shown that paraptosis has been demonstrated to be dependent on mitogen-activated protein kinase (MAPK) family members, and it can be inhibited by AIP-1/Alix (111). Only a few studies have shown a relationship between TP53 and paraptosis. Binghui Li (112)found that ginsenoside RH2 induces apoptosis and paraptosis-like cell death in colorectal cancer cells through activation of TP53.

## Clinical intervention for TP53 mutation

As a transcription factor, p53 mainly regulates cell cycle arrest and apoptosis after activation. TP53 regulates the cell cycle and apoptosis by transcriptional activation of p21/WAF1. P21 binds to cyclin E/CDK2 and the cyclin D/CDK4 complex, with the result that the cell cycle remains at G1 phase (113–115). A tumor with TP53 mutation loses control of the cell cycle, responds poorly to anti-tumor therapy, and has a bleak prognosis (116–118). Therefore,clinical malignant tumors are often accompanied by mutations of TP53. For example, the mutation rate of TP53 in triple negative breast cancer is 80% (119). Triple-negative breast cancer has a very high rate of metastasis and recurrence in clinical practice, and also shows strong resistance to radiotherapy and chemotherapy (120). In clinical treatment of these tumors, it is often necessary to develop new strategies. Therefore, the study of targeted drugs for TP53 mutations provides a new way for clinical treatment.

There are three potential treatment strategies for mutant p53, namely, prevention of degradation of wild-type p53, inhibition of TP53 mutation, and recovery of the function of mutant p53 (121, 122). Agents that protect wild-type p53, such as Nutlin3a, inhibit development of tumors by interfering with negative regulators of p53, especially MDM2 (123). Various strategies designed to recover the function of p53 function are being developed based on the diverse structure and specific functional deficiencies of mutant p53 (124). For example, PRIMA-1 (125), PK11007 (126), and ZMC1 (127) can recover the specific DNA binding sequence and transactivate the p53 target gene by stabilizing the natural structure of the core region of p53 (**Table 1**).

**Table 1:** Agents that target TP53 mutation.

**Compound**	**Mechanism**	**References**
**PRIMA-1**	restore the wild-type conformation to mutant P53 and induce apoptosis in cancer cells	(128)
**APR-246(PRIMA-1^Met^)**	restore the wild-type conformation to mutant P53 and induce apoptosis in cancer cells	(129, 130)
	reduce glutathione(GSH) and thioredoxin reductase 1 (TXNRD1)	
	increase ROS levels	
**COTI-2**	promote refolding of mutant p53 and restore wild-type-p53 function	(124, 131)
	lead to activation of AMPK and inhibit the PI3K-AKT pathway	
**MIRA1**	restore transcriptional transactivation to mutant p53 in living cells	(132)
**STIMA-1**	preferentially kill mutant p53‐carrying tumor cells	(133)
	activate caspases and induces Bax, PUMA and p21	
**Zinc metallochaperone-1 (ZMC1/NSC319726)**	activate mutant p53 by restoring proper zinc loading	(134, 135)
	decrease cellular GSH levels and increase ROS levels	
**PK11007 and other similar compounds(PK11000,PK11010,PK11029,PK11003,PK11012 and PK11015)**	alkylation of surface-exposed cysteines 182 and 277 and stabilized the p53 DBD without impairing its DNA-binding affinity	(126)
	increase protein and mRNA levels of the p21 and PUMA	
	decrease cellular GSH levels and increase ROS levels	
**ReACp53 and related peptides(CDB3)**	disrupts mutant-p53 aggregates and stabilise wild-type conformation	(136–138)
**CP-31398**	protect p53 from thermal degeneration,restore the wild type function of some mutant p53 and up-regulate p53 levels	(139, 140)
**PhiKan083(PK083)**	Binds to the DNA-binding domain of mutant-p53 and restore the wild type function of some mutant p53	(141–143)
**RETRA**	Treatment of mutant p53-expressing cancer cells with RETRA results in a substantial increase in the expression level of p73	(144)
**PC14586**	stabilize the Y220C mutant and restore p53 wild-type (normal) conformation	(145)
**MDM2 Inhibitor:Nutlin-3 and ALRN-6924**	Increase p53 levels and activity	(146, 147)
**RG7388 and AMG232**	disrupt the p53-MDM2 protein–protein interaction and prevent p53 from proteasomal degradation	(148, 149)

## Clinical detection of TP53 mutation

DNA sequencing is regarded as the gold standard for identification of TP53 mutation in a tumor. TP53 mutation is generally located in exons 5–8 of the DNA conservation sequence, but it is as yet a limited area of research. Sanger and high-throughput sequencing are used in both the laboratory and clinical settings to detect mutated TP53, and sequencing analysis before treatment helps to ensure that the therapy provided is appropriate (150, 151). However, although Sanger sequencing is a simple and readily accessible technology, it is insensitive, and second-generation sequencing is able to detect low-frequency mutations below the Sanger sequencing threshold.

**Figure 9:**
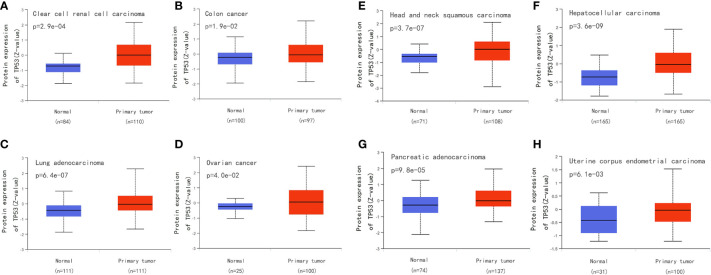
Compare the p53 level between normal and primary tumor tissues for eight types of cancer from the Clinical Proteomic Tumor Analysis Consortium dataset in the UALCAN database (http://ualcan.path.uab.edu/). **(A)** Compare the p53 level between normal and primary tumor tissues for clear cell renal cell carcinoma. **(B)** Compare the p53 level between normal and primary tumor tissues for colon cancer. **(C)** Compare the p53 level between normal and primary tumor tissues for Lung adenocarcinoma. **(D)** Compare the p53 level between normal and primary tumor tissues for ovarian cancer. **(E)** Compare the p53 level between normal and primary tumor tissues for head and neck squamous carcinoma. **(F)** Compare the p53 level between normal and primary tumor tissues for hepatocellular carcinoma. **(G)** Compare the p53 level between normal and primary tumor tissues for pancreatic adenocarcinoma. **(H)** Compare the p53 level between normal and primary tumor tissues for uterine corpus endometrial carcinoma.

Surgical tissue specimens, cell lines, and blood samples can be used to detect TP53 mutation. Tumor DNA can also be studied under non-invasive conditions using cell-free DNA, which is segmented DNA obtained from cells circulating in the blood. There is some evidence showing that the total amount of circulating DNA in patients with cancer is higher than that in healthy subjects (152). In addition to direct molecular analysis of TP53 gene mutations, overexpression of p53 is often used as an alternative marker for abnormalities. We compared the p53 level between normal and primary tumor tissues for eight types of cancer from the Clinical Proteomic Tumor Analysis Consortium dataset in the UALCAN database(http://ualcan.path.uab.edu/) and found higher expression of p53 in tumor tissue (**Figures 9A–H**). However, there are TP53 mutations without overexpression of p53, which may be explained by frameshift mutations leading to truncated proteins that cannot be detected by immunohistochemistry. Therefore, to be able to conduct more detailed research on TP53 mutation, it is necessary to optimize the sequencing technology and standardize the methods used to assess pathogenicity (18).

At present, the correlation between TP53 germline variations and Li-Fraumeni Syndrome (LFS) has been confirmed clinically, and “Chompret criteria” has been established for LFS. It is estimated that at least 20% of persons who meet the Chompret criteria show a detectable variation in the pathogenicity of TP53 (153). At the same time, the screening criteria for the TP53 Chompret criteria were updated in 2015 (154). In addition to LFS, the status of TP53 in chronic lymphocytic leukemia (CLL) has also received attention. Researchers representing the European Research Initiative on CLL (ERIC) made recommendations for the analysis of the TP53 mutation within the LLC (155). In addition, the TP53 mutation can be used as an indicator of poor prognosis in non-small cell lung cancer, particularly in patients with adenocarcinoma and stage I (156). Similarly, TP53 mutations are common in breast cancer, particularly in triple-negative breast cancer where the rate of TP53 mutations can reach 80% (119). And TP53 mutations were associated with increased mortality inpatients with luminal B, HER2-enriched, and normal-like tumors (157). A growing number of drugs aimed at TP53 mutations are also used clinically (**Table 1**).

## Conclusions

PCD is an important process for maintaining the dynamic balance of organisms and can lead to shrinkage of tumors. TP53 is an important tumor suppressor gene and has an important regulatory role in PCD. However, TP53 mutations often occur in cancer cells and interfere with regulation of PCD. Cancer cells can evade these death mechanisms, which is likely to cause tumor growth and metastasis. Therefore, exploration of the role of mutant genes in the development of cancer is very important in terms of its treatment. At present, the mechanisms of TP53 mutation in apoptosis, ferroptosis, and autophagy are relatively clear but require further research in pyroptosis, NETosis, cuproptosis, and immunogenic cell death.

## Author contributions

JW and YLS proposed, wrote, and edited the manuscript. YLS prepared and drew the table and figures accompanying the manuscript. YYS, LZ, ZL, PD, and JZ critically reviewed the manuscript. All authors read and approved the final draft of the manuscript submitted for publication.

## Funding

This work was supported by grants from the National Natural Science Foundation of China (82173065).

## Acknowledgments

We thank Liwen Bianji (Edanz) (www.liwenbianji.cn) for editing the English text of a draft of this manuscript.

## Conflict of interest

The authors declare that the research was conducted in the absence of any commercial or financial relationships that could be construed as a potential conflict of interest.

## Publisher’s note

All claims expressed in this article are solely those of the authors and do not necessarily represent those of their affiliated organizations, or those of the publisher, the editors and the reviewers. Any product that may be evaluated in this article, or claim that may be made by its manufacturer, is not guaranteed or endorsed by the publisher.

## Abbreviations

PCD, programmed cell death; ROS, reactive oxygen species; GPX4, glutathione peroxidase 4; SLC7A11, solute carrier family 7 member 11; xCT, cystine-glutamate antiporter; ALOX15, arachidonate 15-lipoxygenase; SAT1, spermidine/spermine N1-acetyltransferase 1; NRF2, nuclear factor erythroid 2-related factor 2; IL, Interleukin; Apaf-1, apoptosis protease activator factor-1; mTOR, mammalian target of rapamycin; DRAM, damage-regulated autophagy modulator; NETs, neutrophil extracellular traps; NADPH oxidase, NOX, nicotinamide adenine dinucleotide phosphate oxidase.
