# Corrigendum: Current insights into the regulation of programmed cell death by TP53 mutation in cancer

**DOI:** 10.3389/fonc.2022.1071831

**Published:** 2022-11-11

**Authors:** Yali Su, Yingying Sai, Linfeng Zhou, Zeliang Liu, Panyan Du, Jinghua Wu, Jinghua Zhang

**Affiliations:** ^1^ Department of Clinical Laboratory, North China University of Science and Technology Affiliated Tangshan Maternal and Child Health Care Hospital, Tangshan, China; ^2^ Department of Clinical Laboratory, North China University of Science and Technology Affiliated Hospital, Tangshan, China

**Keywords:** TP53 mutation, ferroptosis, pyroptosis, apoptosis, autophagic cell death, cancer

In the published article, there was an error in **Table 1** as published. The order of the references in **Table 1** was incorrect. The corrected **Table 1** appears below.

**Table 1:** Agents that target TP53 mutation.

Compound	Mechanism	References
**PRIMA-1**	restore the wild-type conformation to mutant P53 and induce apoptosis in cancer cells	(128)
**APR-246(PRIMA-1^Met^)**	restore the wild-type conformation to mutant P53 and induce apoptosis in cancer cells reduce glutathione(GSH) and thioredoxin reductase 1 (TXNRD1) increase ROS levels	(129) (130)
**COTI-2**	promote refolding of mutant p53 and restore wild-type-p53 function lead to activation of AMPK and inhibit the PI3K-AKT pathway	(124, 131)
**MIRA1**	restore transcriptional transactivation to mutant p53 in living cells	(132)
**STIMA-1**	preferentially kill mutant p53‐carrying tumor cells activate caspases and induces Bax, PUMA and p21	(133)
**Zinc metallochaperone-1 (ZMC1/NSC319726)**	activate mutant p53 by restoring proper zinc loading decrease cellular GSH levels and increase ROS levels	(134, 135)
**PK11007 and other similar compounds(PK11000,PK11010,PK11029,PK11003,PK11012 and PK11015)**	alkylation of surface-exposed cysteines 182 and 277 and stabilized the p53 DBD without impairing its DNA-binding affinity increase protein and mRNA levels of the p21 and PUMA decrease cellular GSH levels and increase ROS levels	(126)
**ReACp53 and related peptides(CDB3)**	disrupts mutant-p53 aggregates and stabilise wild-type conformation	(136-138)
**CP-31398**	protect p53 from thermal degeneration,restore the wild type function of some mutant p53 and up-regulate p53 levels	(139, 140)
**PhiKan083(PK083)**	Binds to the DNA-binding domain of mutant-p53 and restore the wild type function of some mutant p53	(141-143)
**RETRA**	Treatment of mutant p53-expressing cancer cells with RETRA results in a substantial increase in the expression level of p73	(144)
**PC14586**	stabilize the Y220C mutant and restore p53 wild-type (normal) conformation	(145)
**MDM2 Inhibitor : Nutlin-3 and ALRN-6924**	Increase p53 levels and activity	(146, 147)
**RG7388 and AMG232**	disrupt the p53-MDM2 protein–protein interaction and prevent p53 from proteasomal degradation	(148, 149)

The authors apologize for this error and state that this does not change the scientific conclusions of the article in any way. The original article has been updated.

## Publisher’s note

All claims expressed in this article are solely those of the authors and do not necessarily represent those of their affiliated organizations, or those of the publisher, the editors and the reviewers. Any product that may be evaluated in this article, or claim that may be made by its manufacturer, is not guaranteed or endorsed by the publisher.

